# Wireless Kitchen Fire Prevention System Using Electrochemical Carbon Dioxide Gas Sensor for Smart Home

**DOI:** 10.3390/s22113965

**Published:** 2022-05-24

**Authors:** Soon-Jae Kweon, Jeong-Ho Park, Chong-Ook Park, Hyung-Joun Yoo, Sohmyung Ha

**Affiliations:** 1 Division of Engineering, New York University Abu Dhabi, Abu Dhabi 129188, United Arab Emirates; 2 System LSI Business, Samsung Electronics Co., Ltd., Hwaseong-si 18448, Korea; jeongho.park@samsung.com; 3 Department of Materials Science and Engineering, Korea Advanced Institute of Science and Technology, Daejeon 34141, Korea; cops@kaist.ac.kr; 4 School of Electrical Engineering, Korea Advanced Institute of Science and Technology, Daejeon 34141, Korea; hjyoo53@kaist.ac.kr; 5 Tandon School of Engineering, New York University, New York, NY 10012, USA

**Keywords:** carbon dioxide sensor, fire alarm, fire safety, gas stove, moving average, on-off keying, wireless communication

## Abstract

This paper presents a wireless kitchen fire prevention system that can detect and notify the fire risk caused by gas stoves. The proposed system consists of two modules. The sensor module detects the concentration of carbon dioxide (CO${}_{2}$) near the gas stove and transmits the monitoring results wirelessly. The alarm module, which is placed in other places, receives the data and reminds the user of the stove status. The sensor module uses a cost-efficient electrochemical CO${}_{2}$ sensor and embeds an in situ algorithm that determines the status of the gas stove based on the measured CO${}_{2}$ concentration. For the wireless communication between the modules, on-off keying (OOK) is employed, thereby achieving a longer battery lifetime of the alarm module, low cost, and simple implementation. To increase the lifetime further, a wake-up function based on passive infrared (PIR) sensing is employed in the alarm module. Our system can successfully detect the on state of the stove within 40 s and the off state within 200 s. Thanks to the low-power implementation, in situ algorithm, and wake-up function, the alarm module’s expected battery lifetime is extended to about two months.

## 1. Introduction

With the rapid development of information and communication technology (ICT) and the internet of things (IoT), the smart home, which integrates network-connected remote sensors and automated appliances, has emerged as a recent trend [1,2]. Self-monitoring and automation systems have been used for various tasks in smart homes, such as environment control, energy management, quality-of-life improvement, and safety improvement [1,2,3]. Among them, safety is one of the most critical purposes of smart home systems because the lack of safety may often result in property damage and even causalities. In particular, fire breakouts often cause significant loss and damage because of the spreading nature of fire [1]. A fire in a building can be caused by various sources, such as lights, candles, electricity, cooking, etc. Among them, cooking is one of the major sources of fire. For example, cooking, along with heating and electrical malfunctioning, were listed as leading causes of fires in buildings in the USA from 2003 to 2016 [4]. Among the fire breakouts that occurred in Taiwan from 2013 to 2018, cooking was listed as the second leading cause [1]. Gas stoves and electric stoves have been widely used for cooking in the kitchen. According to 2019 statistics from the United States Census Bureau, although electric stoves are used more in recently built houses, about 37.9% of households in the USA still use gas stoves, using natural or propane gas [5]. In other words, about 49 million households in the USA still use gas stoves. As such, a significant number of households currently use gas stoves. It is always useful to have CO${}_{2}$ sensors or sensors for other high-risk gases, for the safety of smart homes. Therefore, this research focuses on a wireless sensor system that provides a fire risk alarm caused by gas stoves in the kitchen, thereby improving the safety of the whole house.

For fire prevention in a kitchen, it is important to monitor and evaluate the status of the kitchen autonomously. Thin plastic discs, which are attached to the stove knob, are commercially available in the market as a kitchen fire prevention system [6]. The plastic disc beeps and blinks at a pre-programmed interval while the knob is turned on. It is low-cost, but the user may miss the alarm if the user leaves the home with the stove on between the pre-programmed intervals. Moreover, the continuous beeping may be unpleasant. Therefore, a more intelligent prevention system is required. In the literature, a few intelligent prevention systems have been proposed. Thermal cameras with image processing have been employed for obtaining thermal images of the kitchen and identifying dangerous situations [7,8]. In addition, thermal images can be used to recognize human activities and monitor the status of other house appliances [8]. As such, this method is considered useful for maintaining the kitchen safety. However, thermal cameras are typically expensive [9], and the image processing requires a large volume of computing resources. Thus, this method would not be suitable for low-cost, low-power devices. Moreover, thermal cameras sometimes make misjudgments on the situation, which can often happen during cooking processes, such as a situation when hot water is placed on the gas stove. Another possible approach for identifying potential causes of fires in the kitchen is a method to detect flames or smoke by using images obtained by optical cameras [10,11,12,13,14]. However, a certain amount of flames and smoke is often created during cooking processes, so it may be difficult to determine the fire risk by observing flames or smoke only. Moreover, flames and smoke may not be detected by this method before a fire breakout.

Smart home systems usually utilize various sensors to monitor the house [15]. Likewise, as an alternative to cameras, kitchen fire detection and prevention systems have also adopted various sensors, such as temperature sensors [1,9,16,17,18,19,20,21,22], humidity sensor [1,9,19,20,21,22], flame sensor [1,3,19], smoke sensor [16], ultrasound sensor [9], gas-leakage sensor [1,22,23,24], carbon monoxide (CO) sensor [16,18,20], carbon dioxide (CO${}_{2}$) sensor [18], and so on. In general, these sensors are cheaper, smaller, more powerful, and cost-efficient than cameras. Thus, they have been widely adopted in many affordable kitchen fire detection and prevention systems. However, methods using temperature and humidity sensors have the same disadvantage as those using thermal images. With temperature and humidity sensors, some situations are difficult to identify, such as a situation where a pot filled with water is placed on the gas stove with the stove turned off. When flame sensors and smoke sensors are employed for identifying dangerous situations in the kitchen, they have the same limitations as the method using images for distinguishing flames or smoke. Flames and smoke may be detected after a fire, not before the fire. In addition, since the flame sensor also monitors light [1], a wrong decision may occur depending on the lighting condition in the kitchen. Ultrasound sensors, which measure the distance between the sensor and the stove, have been used to identify the absence of an object on the stove. However, detection of objects on the stove cannot tell whether it is safe or not. Two types of gas sensors have been adopted for kitchen fire prevention. Gas-leakage sensors, which are sensitive to fuels of gas stoves, such as methane, propane, butane, and natural gas, have been adopted for detecting leakage of combustible gases, rather than detecting fire risks caused by the on state of the gas stove. In contrast, CO sensors can measure the concentration of CO generated by the combustion process so that they can be used to detect the status of the gas stove and identify the fire risk during cooking processes. However, as shown in the results of field studies with gas stoves in the kitchen, the highest concentration of CO measured during one hour in the kitchen does not exceed about 30 ppm [25] and the highest concentration of CO measured during four hours in the kitchen does not exceed about 20 ppm [26]. The CO concentration is typically very low during the combustion process. Thus high-resolution sensors may be needed to measure the concentration and determine the status based on the measured results.

Alternatively, this paper presents a wireless kitchen fire prevention system for improving the safety of smart homes by monitoring CO${}_{2}$ concentration. It evaluates the fire risk caused by gas stoves and notifies the user. It has been reported that the CO${}_{2}$ concentration varies up to 2500 ppm during cooking processes [26]. Such concentration variation of CO${}_{2}$ is much larger than that of CO. Thus, the CO${}_{2}$ sensor is not required to have high resolution and sensitivity compared to the case using CO sensors. In addition, the proposed prevention system can avoid malfunctions caused by non-combustible objects, i.e., a pot filled with water or an object on the stove. Although a CO${}_{2}$ sensor has already been adopted in a previous system [18], they adopted a non-dispersive infrared (NDIR) CO${}_{2}$ sensor, of which the cost is high. In addition, the system in [18] requires complicated algorithms that are operated on a central hub, such as a computer.

The proposed system consists of two modules: sensor and alarm modules. The sensor module measures the concentration of CO${}_{2}$ generated by the combustion process and determines the on/off status of the stove based on the concentration in situ without using a high-performance computing unit. Instead of transmitting raw data of the CO${}_{2}$ concentration all the time, the data rate of the wireless transmitter in the sensor is reduced by transmitting minimum data bits only. The alarm module, which is operated on a battery, wirelessly receives the status information and reminds the user, which is in a different location, of the status. Thanks to the wireless communication, the gas stove status can be notified remotely for the user not to forget turning off the gas stove. On–off keying (OOK) is adopted for wireless communication for a longer battery lifetime of the alarm module. In order to increase the lifetime further, a wake-up function based on passive infrared (PIR) sensing is employed in the alarm module, turning on the circuits when necessary. The direct application of this work is just to check the status of the gas stove, but its general platform can be used and expanded as a part of smart homes with more diverse sensors and advanced algorithms. This work serves as an initial stepping stone towards a more advanced fire prevention system for smart homes.

The rest of the paper is organized as follows. The overall architecture of the proposed system is explained in Section 2. The detailed implementation is described in Section 3. Section 4 shows the experimental results, with a discussion. Section 5 concludes the paper.

## 2. Proposed System

**Figure 1:**
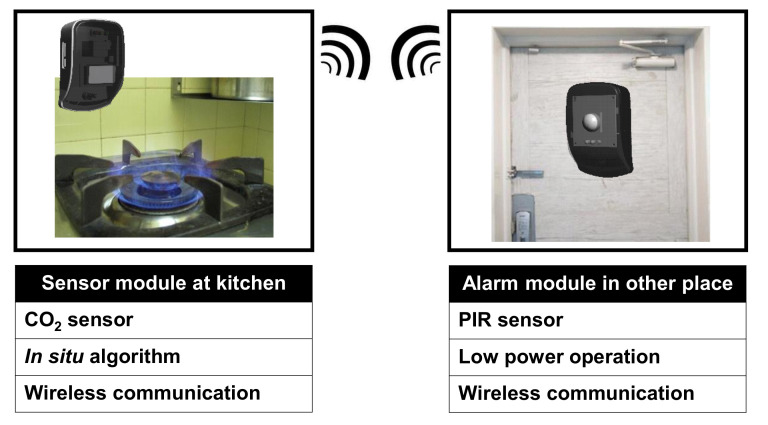
Proposed wireless kitchen fire prevention system for smart home safety.

When a kitchen fire prevention system detects a fire risk, there are two possible methods to prevent the fire: (1) automatic turn-off of the stove and (2) a reminder to the user [3]. The automatic turn-off method could ensure safety, but it often causes inconveniences in some situations while cooking. For example, when the users need to let food simmer for a long time, the automatic turning-off of the stove function could disturb the intended cooking process [3]. To avoid this issue, our proposed wireless kitchen fire prevention system sends a reminder to the user, as shown in Figure 1. The system has a sensor module that monitors the gas stove in the kitchen and sends data to a remotely placed alarm module to alarm the user. The transmitter in the sensor module transmits the status of the kitchen to the receiver in the alarm module through wireless communication and, thus, the user can make a decision on what to do, e.g., turn off the stove when needed.

Liquefied petroleum gas (LPG) and liquefied natural gas (LNG) are widely used as fuels for kitchen gas stoves. The composition of LPG is variable, but its major constituent gases of LPG are propane (C${}_{3}$H${}_{8}$) and butane (C${}_{4}$H${}_{10}$) [27]. The main constituent of LNG is methane (CH${}_{4}$) [28]. The chemical formulas for the combustion process of C${}_{3}$H${}_{8}$, C${}_{4}$H${}_{10}$, and CH${}_{4}$ are expressed as follows:

(1) $$C_{3}H_{8}+5O_{2}->3CO_{2}+4H_{2}O,$$

(2) $$2C_{4}H_{1}0+13O_{2}->8CO_{2}+10H_{2}O,$$

(3) $$CH_{4}+2O_{2}->CO_{2}+2H_{2}O.$$

All the gases produce CO${}_{2}$ during the combustion. In the proposed system, CO${}_{2}$ concentration is measured by an electrochemical CO${}_{2}$ sensor placed in the sensor module, which is installed near the gas stove. When the gas stove is turned on, the CO${}_{2}$ concentration increases due to the continuous combustion. Based on the measured concentration, an algorithm determines the on/off state of the gas stove in situ and notifies the user of the alarm when the gas stove is on. Since the absolute concentration can vary by the ventilation condition, it is difficult to determine the status with a single threshold. Hence, the in situ algorithm evaluates the gas stove status by using not only the absolute concentration, but also the change of the concentration. Instead of transmitting the raw data of the CO${}_{2}$ concentration all the time, the wireless transmitter of the proposed system transmits minimum data bits, which are the ID with a preamble, check-bit, and postamble, only when the fire risk is high in an event-driven manner. The remotely placed alarm module just checks the presence of the transmitted data. Since the transmitted data are reduced, the data rate can be increased by about 2.4 times. Moreover, thanks to the event-driven transmission, the power consumption of the receiver can also be reduced because it does not need to operate all the time. The OOK modulation, which is one of the simplest modulation methods, is adopted for wireless communication so that the power consumption and cost of the wireless transmitter and receiver can be further lowered.

The fire risk when the user is far from the gas stove is much greater than that when the user is near the gas stove. The alarm module can be installed in a remote location where the user can timely check the status of the stove. For example, it is very dangerous if the user forgets to turn off the gas stove when leaving the home. Therefore, if the receiver is placed at the house’s main door, the user can easily check the status of the stove and prevent a fire that may be caused by keeping the gas stove turned on while gone. The alarm module operates on a battery, so it can be placed wherever the user wants. Its maximum operating time has to be long enough to avoid the inconvenience of frequent battery replacement. Therefore, the power consumption of the module should be as low as possible. In this regard, adopting OOK is beneficial. Furthermore, it is not power efficient to operate the alarm module all the time even when the user cannot recognize the indicator of the alarm module. In order to reduce the power consumption even more, a wake-up algorithm is employed in the alarm module. A passive infrared (PIR) sensor is used to generate a wake-up signal, turning on the major parts of the module, only when people come near the receiver. The alarm module displays the status only when the PIR sensor detects the presence of the user around the receiver. The maximum operation time is further prolonged by turning off the major parts, such as the microcontroller (MCU) and indicator when the user is far away from the receiver, or any notification is not required.

## 3. Implementation Details

This section presents the design details of the proposed wireless kitchen fire prevention system. The hardware and in situ algorithms of the sensor module, the hardware and wake-up algorithm of the alarm module, and their wireless communication, are explained in more detail.

### 3.1. Sensor Module

**Figure 2:**
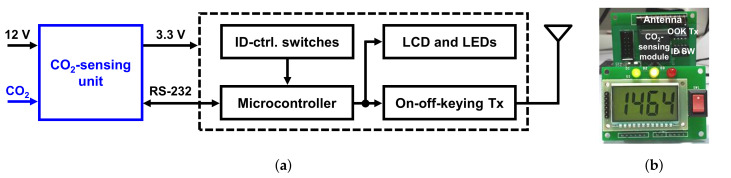
(**a**) Block diagram and (**b**) implemented prototype of the sensor module.

Figure 2 shows a block diagram and the implemented prototype of the sensor module. The module consists of a CO${}_{2}$-sensing unit (CVC-2001, CIOS Inc., Singapore), an MCU (ATMEGA16L, Atmel, San Jose, CA, USA), an OOK transmitter (TXM-433, LINX, Singapore), ID-control switches, and indicators. The module is powered by a 12-V supply voltage converted from the household supply voltage of 220 V by a power adaptor to monitor CO${}_{2}$ concentration all the time. A low-dropout regulator incorporated in the CO${}_{2}$-sensing unit provides a 3.3-V supply voltage to the rest of the building blocks.

The CO${}_{2}$-sensing unit measures CO${}_{2}$ concentration in real-time using an electrochemical CO${}_{2}$ sensor. The operation principle of electrochemical gas sensors is well reviewed in [29,30]. In general, electrochemical gas sensors can be classified into three types according to their operation principles: Type-I, Type-II, and Type-III sensors. Among them, there are only few Type-I and Type-II CO${}_{2}$-sensing devices due to lack of suitable solid electrolytes [29]. Therefore, Type-III sensors are more suitable for sensing CO${}_{2}$ compared to other types. Type-III sensors consist of a sensing electrode, a reference electrode, and solid-state electrolyte between the two electrodes. An auxiliary phase (AP) is employed between the solid electrolyte and the sensing electrode, or the AP may also directly work as the sensing electrode itself. When Na${}_{2}$CO${}_{3}$ is adopted as the AP, the sensing reaction on the surface between the sensing electrode and electrolyte is expressed as follows [29,30]:

(4) $$2Na^{+}+CO_{2}+1/2O_{2}+2e^{-}<->Na_{2}CO_{3}.$$

On the surface between the reference electrode and electrolyte, the reference reaction occurs with oxygen, not with carbon dioxide, as follows [29,30]:

(5) $$2Na^{+}+1/2O_{2}+2e^{-}<->Na_{2}O.$$

A voltage difference between the two electrodes is generated by these two reactions, and the CO${}_{2}$ concentration can be calculated using the Nernst equation with the voltage. The materials of the AP and solid-state electrolyte influence various sensor characteristics, i.e., cost, long-term stability, and so on and, thus, various materials have been used.This electrochemical sensor type is chosen because it is cheaper compared to NDIR type [31].

Table 1 summarizes the performance of the CO${}_{2}$-sensing unit. The sensing unit can measure CO${}_{2}$ concentration from 300 to 5000 ppm with ±20% accuracy. This type of electrochemical sensor has a limited lifetime due to the chemical reactions. The maximum lifetime provided by the manufacturer is 5 years.

The measured concentration data are delivered to the MCU’s memory through the RS-232 protocol every 1.7 s. Based on the data, the MCU determines the ignition state of the gas stove. By using this in situ algorithm, the transmitter does not need to transmit the concentration data all the time, it only transmits ID data with peripheral bits when the gas stove is turned on. Compared to incorporating the algorithm on the receiver side, the amount of transmission data can be reduced by having it on the transmitter side. Thanks to the low data rate, OOK modulation, which is the simplest modulation method, can be adopted for wireless communication. Moreover, the power consumption of the wireless transceivers can be lowered. Furthermore, since the sensor module does not operate on a battery and has less restriction on the power consumption, it is more appropriate to incorporate the algorithm on the transmitter side than on the receiver side. Moreover, the ID-control switches set the transmitter’s unique ID. The transmitter communicates only with the receiver having the same ID. A liquid crystal display (LCD) and light-emitting diodes (LEDs) are adopted as indicators so that the user can monitor the concentration and fire risks near the gas stove as well.

**Figure 3:**
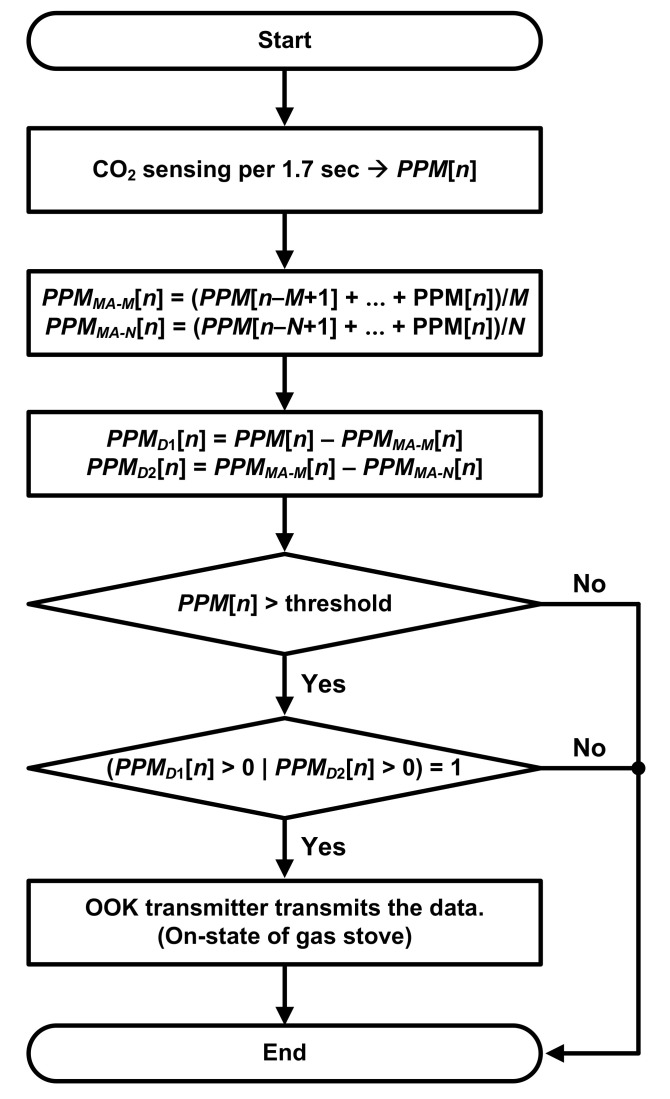
Flow chart of the in situ algorithm that determines the state of the stove.

Figure 3 shows a flow chart of the in situ algorithm. The MCU stores the concentration data, PPM[*n*], which are delivered from the CO${}_{2}$-sensing unit. Two moving-averaged data, $PPM_{\mathrm{MA}-M}$[*n*] and $PPM_{\mathrm{MA}-N}$[*n*], are generated by averaging *PPM*[*n*]. $PPM_{\mathrm{MA}-M}$[*n*] is obtained by averaging consecutive *M* samples, and the $PPM_{\mathrm{MA}-N}$[*n*] is obtained by averaging consecutive *N* samples where *M* > *N*. These moving-average (MA) functions attenuate random noise on the CO${}_{2}$ concentration data through their inherent low-pass filtering, thereby reducing wrong decisions caused by fluctuations from the external environment. $PPM_{D1}$[*n*] and $PPM_{D2}$[*n*] are obtained by taking differences as follows:

(6) $$PPM_{D1}=PPM\left[n\right]-PPM_{\mathrm{MA}-M}\left[n\right],$$

(7) $$PPM_{D2}=PPM_{\mathrm{MA}-M}\left[n\right]-PPM_{\mathrm{MA}-N}\left[n\right],$$

$PPM_{D1}$[*n*] and $PPM_{D2}$[*n*] are used to observe changes in the concentration over time.

The algorithm determines the on/off state of the gas stove with two steps based on (1) the absolute value and (2) the difference values. The first step is to compare the current concentration, PPM[*n*], with a threshold value. When the gas stove is turned on, PPM[*n*] is increased due to the combustion process. When PPM[*n*] is smaller than the threshold value, the algorithm concludes with the off state and is ended for this turn without transmitting any data through the OOK transmitter. Otherwise, the second step is performed when PPM[*n*] is higher than the threshold value. In this step, the algorithm decides the status of the gas stove using $PPM_{D1}$[*n*] and $PPM_{D2}$[*n*]. When either $PPM_{D1}$[*n*] or $PPM_{D2}$[*n*] is positive, the algorithm concludes that the gas stove is turned on. When the CO${}_{2}$ concentration is ramping up, PPM[*n*] is normally higher than $PPM_{\mathrm{MA}-M}$[*n*] because $PPM_{\mathrm{MA}-M}$[*n*] averages the current data with the past data. Similarly, when CO${}_{2}$ concentration is increasing, $PPM_{\mathrm{MA}-N}$[*n*] is higher than $PPM_{\mathrm{MA}-M}$[*n*] because $PPM_{\mathrm{MA}-M}$[*n*] includes more past data compared to $PPM_{\mathrm{MA}-N}$[*n*]. Note that *M* > *N* here. Therefore, if either $PPM_{D1}$[*n*] or $PPM_{D2}$[*n*] is larger than zero, the CO${}_{2}$ concentration is increasing, meaning that the stove is on. Then, the OOK transmitter transmits the ID data with peripheral bits for notifying the fire risk to the receiver.If the algorithm makes decisions on the state by using $PPM_{D1}$[*n*] only, fluctuations caused by noises may disturb the decision, degrading the accuracy. Therefore, $PPM_{D2}$[*n*], which is taken from the two averaged values, is also used for more accurate detection.

### 3.2. Alarm Module

**Figure 4:**
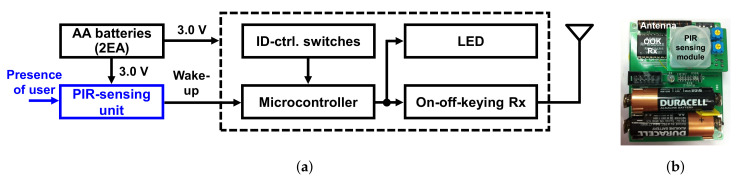
(**a**) Block diagram and (**b**) implemented prototype of the alarm module.

Figure 4 shows a block diagram and the implemented prototype of the alarm module. The module consists of a PIR-sensing unit with a PIR sensor (RE200B, NiCERA), an MCU (ATMEGA16L, Atmel), an OOK receiver (RXM-433, LINX), ID-control switches, an LED indicator, and two AA-size batteries. The MCU controls the overall operation of the module. The ID-control switches set the receiver’s unique ID that matches the transmitter’s one. The PIR-sensing unit detects the presence of users around the module. The PIR-sensing unit’s output is used as the wake-up signal of the whole module, turning on and off the MCU and LED. This event-driven operation reduces the power consumption significantly, extending the lifetime of the battery-powered alarm module. The LED is activated when the wake-up signal is turned high, meaning that a user is nearby. Then, depending on the received data on the status of the gas stove, the LED can be turned on or be kept off.

**Figure 5:**
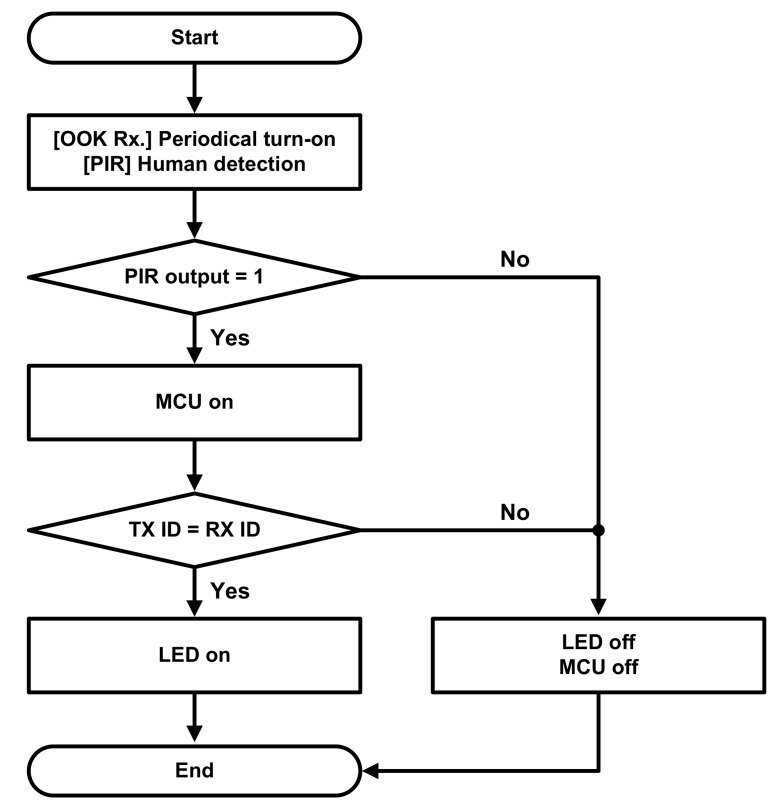
Operation flow chart of the alarm module.

Figure 5 shows an operation flow chart of the alarm module. In order to install the alarm module in any place with a maximized battery life, the MCU operates mostly on the sleep mode with minimal power consumption while the OOK receiver is only turned on periodically. The PIR-sensing unit, which operates all the time, keeps monitoring the presence of users around the module. When the PIR-sensing unit detects a user around the module, it generates a wake-up signal, turning on the MCU. Then, the LED indicator is turned on if the gas stove is turned on. This notifies the user, who may be about to leave home, of the on status of the stove.

### 3.3. Wireless Communication

**Figure 6:**
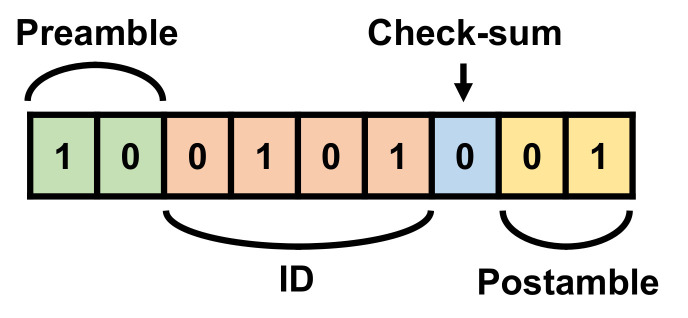
Bit configuration of the data symbol.

The OOK transmitter and receiver communicate with each other using a 433-MHz carrier frequency, which is an ISM band. In order to ensure reliable communication, a transmitted data symbol is configured as shown in Figure 6. The first two bits and last two bits are designated as the preamble and postamble, respectively, for identification of the presence of the communicated data and synchronization between the transmitter and receiver. The symbol also includes a 4-bit ID and 1-bit checksum in between. When the ID in the received data matches with the receiver’s ID, the receiver starts to read the data. The checksum bit tells whether there is an error in the received data. The sum of the ID data and checksum bit is designed to be an odd number. The receiver examines the error by checking whether the sum is odd. Each bit is generated using Manchester encoding with a 4-ms period. Therefore, the symbol period is 36 ms in total. The transmitter sends the data symbol; it does not include any concentration data when it detects the state of the gas stove. Since the number of bits for one data symbol is reduced from 22 to 9 bits, the data rate is increased by about 2.4 times.

The communication distance ($d_{comm}$) is an important parameter of the system. For calculating the maximum $d_{comm}$, a free-space path loss ($P_{FSPL}$) needs to be calculated, and it is expressed as follows:

(8) $$P_{FSPL}=20\log_{10}\frac{4\pi d_{comm}f_{c}}{c}-G_{TX,A}-G_{RX,A},$$

where $f_{c}$ is the carrier frequency, *c* is the speed of light in vacuum, $G_{TX,A}$ is the gain of the transmitting antenna, and $G_{RX,A}$ is the gain of the receiving antenna. When $f_{c}$ = 433 MHz, $d_{comm}$ = 10 km, and $G_{TX,A}=G_{RX,A}$ = −3 dB, $P_{FSPL}$ is about 111.2 dB. When the difference between the output power of the wireless transmitter ($P_{TX}$) and the sensitivity of the wireless receiver ($P_{SENS}$) is larger than the $P_{FSPL}$, the receiver can detect the data transmitted from the transmitter. $P_{TX}$ of the OOK transmitter (TXM-433, LINX) can be controlled from −80 to 10 dBm, and the $P_{SENS}$ of the OOK receiver (RXM-433, LINX) has very low sensitivity of −112 dBm. Therefore, the wireless prevention system can support $d_{comm}$ up to 10 km in ideal environments. Even considering obstacles and design margins, it can sufficiently cover a general house.

## 4. Experimental Results and Discussion

This section presents the experimental results obtained with the prototype of the proposed wireless kitchen fire prevention system. The prototype was installed near a gas stove to validate the system and algorithms. The battery-powered alarm module was placed remotely, communicating with the sensor module wirelessly.

### 4.1. System Operation Tests

**Figure 7:**
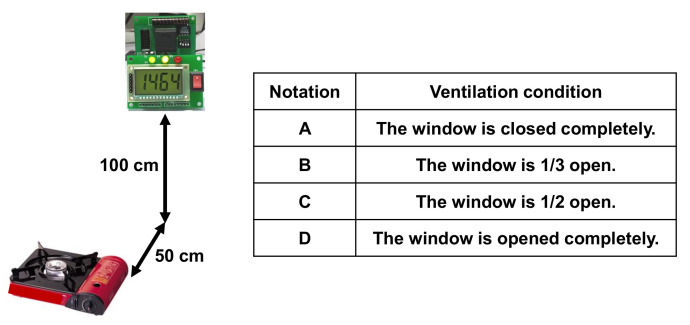
Test setup and ventilation conditions.

**Figure 8:**
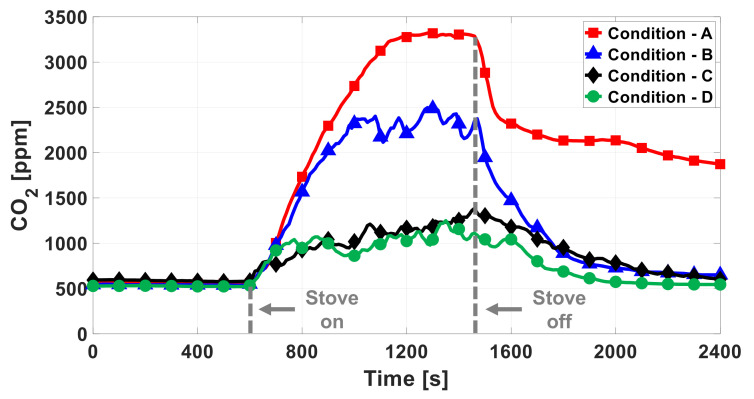
Measured CO${}_{2}$ concentration in the various ventilation conditions in Figure 7. The stove is turned on at 600 s and off at 1500 s.

Figure 7 shows the experimental setup and ventilation conditions for the following tests. The sensor module was installed horizontally, about 50 cm apart, and vertically, about 100 cm apart, from a gas stove. Figure 8 shows the measured CO${}_{2}$ concentrations for each of the four ventilation conditions described in Figure 7. As shown, the CO${}_{2}$ concentration increases significantly when the ventilation is poor. The less the ventilation, the greater the concentration difference between the initial state (before 600 s in Figure 8) and the gas-stove’s on state (between 600 and 1500 s).

It is not suitable to determine the on/off state by only using the first step of the in situ algorithms described in Figure 3, which compares the current concentration with a threshold value. In order to determine the on state quickly, the threshold has to be set to the value just above the initial concentration value. However, by using the method to determine the off state, a fairly long delay would be required. As shown by the measured results in poor ventilation conditions in Figure 8, it takes a long time to recover, back to the initial concentration after the gas stove is turned off. In particular, the worse the ventilation, the longer the recovery.

**Figure 9:**
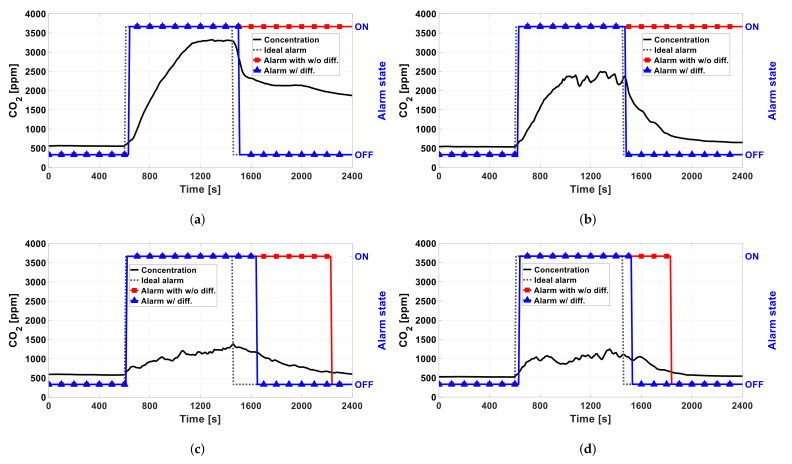
Measured CO${}_{2}$ concentration and the alarm state determined by the algorithm for the ventilation conditions described in Figure 7: (**a**) condition A, (**b**) condition B, (**c**) condition C, and (**d**) condition D. The dashed lines indicate the actual on/off times of the stove. The red lines are the alarm state determined by the threshold-based method, while the blue lines are the alarm state determined by the proposed algorithm using the absolute and difference values together.

Figure 9 shows the processed state obtained by the in situ algorithm along with the measured CO${}_{2}$ concentration for each ventilation condition. It also shows the processed state that is obtained by using the threshold-based first step only without the difference-based second step. In the figures, the dashed line indicates the actual timings of turning on and off the stove. Table 2 summarizes the measured delay times between the actual on/off timings and the detected timings ($T_{D,ON}$ and $T_{D,OFF}$). $T_{D,ON}$ is the delay between the stove’s actual turn-on time and the receiver’s on notification. $T_{D,OFF}$ is the delay between the stove’s actual turn-off time and the receiver’s off notification. The absolute threshold concentration was set to 650 ppm to detect the on state of the stove quickly. *M* and *N*, which is the moving-averaging factors of the in situ algorithm, were set to 8 and 3, respectively, in order to avoid wrong decisions due to the fluctuations. The threshold, *M*, and *N* values were determined by optimization based on the experiment results. In this work, the in situ algorithm was run on a very simple micro-controller, so such simple algorithms only could be used. When more computing resources are available, more complicated algorithms with adaptive parameters may be used, even with machine learning. With the implemented algorithm, false alarms may occur due to other sources, which increase the CO${}_{2}$ concentration. However, a high concentration or dramatic increase of CO${}_{2}$ indicates poor air quality, which may be harmful to health, so it is necessary to notify the user for both safety and health. As shown in Figure 9 and Table 2, our system can successfully detect the on state of the stove within 40 s and the off state within 200 s. Compared to the threshold-based method, $T_{D,OFF}$ is significantly shortened by utilizing the absolute and difference values together.

It was validated that the alarm works correctly in various ventilation situations and, thus, the CO${}_{2}$ concentration can be used as an indicator to determine the state of the gas stove. However, it has some limitations. The ideal *M* and *N* parameters, which determine the length of the moving averages in the detection algorithm, may need to be changed according to the external environment, especially the ventilation condition, including the position and size of the window and the wind condition. Moreover, CO${}_{2}$ measurements typically fluctuate, which may lead to wrong decisions. Furthermore, the diffusion of CO${}_{2}$ is slow, limiting the speed of the CO${}_{2}$-based fire detection, so it results in long $T_{D,ON}$ and $T_{D,OFF}$. This slow response could be improved by adopting different kinds of sensors. Among the various sensors in previous works, temperature and humidity sensors do not have rapid responses, such as the CO${}_{2}$ concentration during the cooking process. Flame sensors adopted in [1,3,19] might be good candidates to shorten $T_{D,ON}$ and $T_{D,OFF}$ because they have a fast response. CO${}_{2}$ sensors are not sensitive to light, while flame sensors are not sensitive to ventilation conditions. Therefore, combining a flame sensor with a CO${}_{2}$ sensor would be synergic to achieve a fast response. Moreover, false positive alarms of CO${}_{2}$ concentration caused by other sources, except gas stoves, can be reduced by using other sensors, such as flame sensors.

According to Figure 9c,d, and Table 2, the alarm works correctly even in poor ventilation conditions where the oxygen level is lower. When oxygen is not enough in the combustion process, a more incomplete combustion process happens, generating CO instead of CO${}_{2}$. In that case, CO${}_{2}$ sensors may fail to detect the fire. Therefore, it would be beneficial to include a CO sensor, such as ones in [16,18,20], together with the CO${}_{2}$ sensor. In addition, if the system also have a gas-leakage sensor, which is adopted in [1,22,23,24], the system can detect gas leakages, further reducing the fire risk. These sensors can be merged to our system easily, and such multi-sensor systems would be a promising future work. CO${}_{2}$, CO, and fuels, such as LPG and LNG, may have fire risks and harmful effects to health, so a similar simple in situ algorithm using both the absolute concentration and the change of the concentration can be applicable to alarm risk of each or all combined. The algorithm itself and its parameters should be developed based on more experiments in the future.

### 4.2. Current Consumption and Battery Life Time

**Figure 10:**
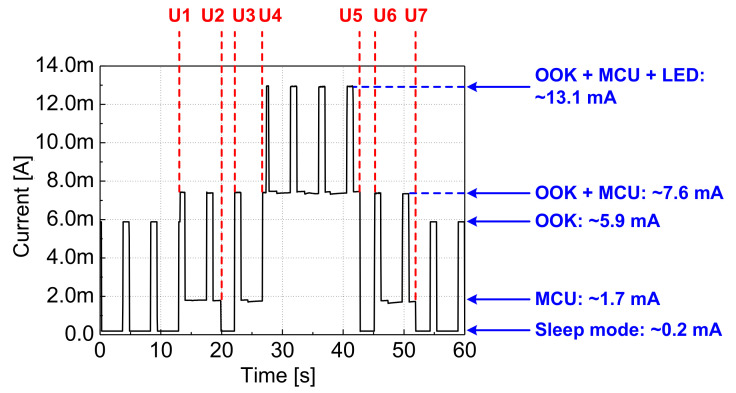
Current consumption of the alarm module.

Figure 10 shows the measured current consumption of the alarm module in various scenarios described in Table 3. When a user is not present around the alarm module, the MCU stays in the sleep mode. The PIR sensor is always turned on, detecting the presence of users. The OOK receiver periodically communicates with the transmitter by using a timer of the MCU in order to check the presence of data from the transmitter. The alarm module consumes only about 0.2 mA when the OOK receiver is turned off and about 5.9 mA during the communication process. When the PIR sensor detects a user, the MCU is turned on and reads the data of the OOK receiver. At that time, the alarm module consumes 7.6 mA. The MCU returns to the sleep mode again when the user leaves the module. On the other hand, when the user stays around the module and the gas stove is in the on state, both the MCU and LED are turned on. In this condition, the alarm module consumes its maximum current of 13.1 mA. As a result, the current consumption can be drastically reduced from 13.1 to 0.2 mA during most of the time when the user is not present around the receiver, thereby extending the battery life.

The average current consumption ($I_{AVG}$) and corresponding battery lifetime ($T_{BAT}$) depend on various conditions, such as the on time of the gas stove and the time that the user stays around the alarm module. $I_{AVG}$ is expressed as follows:

(9) $$\begin{array}{c} I_{AVG}=0.05\times 13.1\;\mathrm{mA}+0.2\times 5.9\;\mathrm{mA}+0.75\times 0.2\;\mathrm{mA}=1.985\;\mathrm{mA}. \end{array}$$

Equation (9) is calculated under the following three assumptions. The first assumption is that the user stays near the receiver while the gas stove is turned on 3 min per hour. Moreover, the OOK receiver operates with a 20% duty cycle. Lastly, the alarm module operates in the sleep mode for the remaining time. With a given 2700-mAh battery capacity, the receiver can operate for about 57 days without battery replacement.

As the duty cycle of the OOK receiver decreases, the expected battery life increases. When the duty cycle is reduced from 20% to 10%, the lifetime increases from 57 to 80 days. However, the latency from the moment that the PIR sensor detects the user to the moment that the LED indicates the status of the gas stove is compromised instead. Moreover, by reducing the duty cycle of the LED control, the current consumption of the LED can also be reduced, thereby prolonging the battery time. When the duty cycle of 20% is used for turning on the LED, the battery lifetime can further increase from 80 days to 94 days.

### 4.3. Price

Table 4 and Table 5 summarize the estimated costs of the sensor module and alarm module, respectively. The cost of electrochemical CO${}_{2}$ sensors is typically lower than other types of CO${}_{2}$ sensors. For example, MG-812 (Winsen Co., Ltd., Singapore) [32] is about half the cost of the NDIR CO${}_{2}$ sensors, such as MH-Z19B (Winsen Co., Ltd.) [33].

## 5. Conclusions

The performances of the proposed kitchen fire prevention system and other kitchen fire prevention systems are summarized and compared in Table 6. The proposed system has four novelties: (1) This system is one of the first kitchen fire prevention systems based on a electrochemical CO${}_{2}$ sensor, which is cheaper than the NDIR type; (2) this system has two units, one for sensing and the other for alarming, and they communicate wirelessly through simple OOK communication; (3) the sensing module embeds an in situ algorithm for reducing the data rate and avoiding the use of other expensive computing unit; (4) the alarm module embeds a wake-up algorithm based on human sensing for low power operation.

This paper presents a wireless kitchen fire prevention system for the smart home safety by notifying the user of the fire risk caused by the gas stove. The proposed system consists of sensor and alarm modules. The sensor module determines the status of the gas stove in the kitchen by using its in situ algorithm based on the measured CO${}_{2}$ concentration. The battery-powered alarm module, which is placed remotely, such as at the house front door, communicates with the sensor module and reminds the user of the status of the gas stove. A low-cost, low-power OOK transmitter and receiver are adopted for wireless communication for a long battery lifetime and low cost. To increase the lifetime further, a PIR sensor of the alarm module continuously monitors the user’s presence around the module, and the MCU operates on sleep mode when the user is not around the receiver. A wake-up algorithm is employed for turning on the MCU and LED only when the user is around the module. Our system can successfully detect the on state of the stove within 40 s and the off state within 200 s in various ventilation conditions.The expected battery lifetime of the alarm module with two AA batteries is about two months.

In this paper, it is successfully demonstrated that this work can evaluate the status of a gas stove through the CO${}_{2}$ sensor and an in situ algorithm and generate a remote alarm through wireless communication. In future research, this work can be easily expanded to a kitchen fire-prevention system that can communicate with smart devices, i.e., a smart phone and watch, through a wireless network already used in smart devices. When connected with smart devices with larger computing resources than the simple MCU that this work uses, more advanced algorithms, such as machine-learning-based ones, can be employed for higher accuracy. In addition, sensor fusion with CO sensors, other gas sensors, and flame sensors would be a future direction of research, for more thorough detection of various fire risks and to improve the detection time, thereby achieving better prevention systems toward future smart homes.

## Figures and Tables

**Table 1:** Performance summary of CO${}_{2}$-sensing unit.

Product	Detection	Accuracy	Operational Temp. &	Lifetime	Power
Name	Range [ppm]	[%]	and Humid Range	[Years]	[W]
CVC-2001	300–5000	±20	0–50 ${}^{\circ }$C, 5–90% RH	≤5	≤1

**Table 2:** On and off delays of the detection.

	Condition—A		Condition—B		Condition—C		Condition—D	
	$T_{D,\mathrm{ON}}$	$T_{D,\mathrm{OFF}}$	$T_{D,\mathrm{ON}}$	$T_{D,\mathrm{OFF}}$	$T_{D,\mathrm{ON}}$	$T_{D,\mathrm{OFF}}$	$T_{D,\mathrm{ON}}$	$T_{D,\mathrm{OFF}}$
Alarm with algo.	40 s	60 s	30 s	30 s	20 s	200 s	40 s	80 s
Alarm with abs.	40 s	Infinity	30 s	Infinity	20 s	790 s	40 s	390 s

**Table 3:** The test scenarios used for measuring the current consumption in Figure 10.

Notation	Situation
U1	The user approaches the alarm module.
U2	The user leaves the alarm module.
U3	The user approaches the alarm module again while the gas stove is turned off.
U4	The user stays around the alarm module, and the gas stove is turned on.
U5	The user turns off the gas stove.
U6	The user approaches the alarm module again, but the gas stove is turned off.
U7	The user leaves the alarm module.

**Table 4:** Estimated cost of the sensor module.

CO${}_{2}$-Sensing	Micro-	Wireless Tx.	Indicators	ID-Ctrl.	Total
Unit ${}^{a}$	Controller	with Antenna	(LCD and LEDs)	Switches	Price
$14.0	$5.0	$3.3	$2.6	$0.4	$25.3

^a^: CVC-2001 is not available in current market. Another electrochemical CO${}_{2}$ sensor (MG-812) [32] is adopted to estimate the price.

**Table 5:** Estimated cost of the alarm module.

PIR-Sensing	Micro-	Wireless Rx.	Indicators	ID-Ctrl.	Total
Unit ${}^{a}$	Controller	with Antenna	(LED)	Switches	Price
$8.0	$5.0	$5.3	$0.1	$0.4	$13.8

^a^: A RE200B-based module developed by Adafruit is used to estimated the price.

**Table 6:** Performance summary and comparison of kitchen fire prevention systems.

	This Work	[1]	[3]	[16]	[18]	[20]
		Temp., humid.,		Temp.,	Temp.,	Temp.,
Sensors	Electrochemical CO${}_{2}$	flame,	Flame	smoke,	CO,	humid.,
		gas-leakage		CO	NDIR CO${}_{2}$	flame, CO
Remote	Yes	Yes	Yes	Yes	Yes	Yes
alarm	(OOK)	(Wi-Fi)	(BLE)	(Zigbee)	(Zigbee, GSM)	(GSM)
Evaluation	in situ	ex situ	ex situ	in situ	ex situ	ex situ
Algorithm	(MCU)	(MCU)	(MCU)	(MCU)	(Central hub)	(Computer)
$T_{D,ON}$ [s]	20–40	∼0	N. R.	N. R.	N. R.	N. R.
$T_{D,OFF}$ [s]	30–200	∼0	N. R.	N. R.	N. R.	N. R.
Wake-up	Human sensing	No	Flame sensing	Duty cycle	No	No

## Data Availability

Not applicable.
